# Retraction Note to: Matrine derivate MASM uncovers a novel function for ribosomal protein S5 in osteoclastogenesis and postmenopausal osteoporosis

**DOI:** 10.1038/s41419-022-04697-w

**Published:** 2022-03-11

**Authors:** Xiao Chen, Xin Zhi, Liehu Cao, Weizong Weng, Panpan Pan, Honggang Hu, Chao Liu, Qingjie Zhao, Qirong Zhou, Jin Cui, Jiacan Su

**Affiliations:** 1 Department of Orthopedics Trauma, Shanghai Changhai Hospital, Second Military Medical University, Yangpu District, Shanghai, 200433 China; 2 China-South Korea Bioengineering Center, Jiading District, Shanghai, 201802 China; 3 Graduate Management Unit, Shanghai Changhai Hospital, Second Military Medical University, Yangpu District, Shanghai, 200433 China; 4 School of Pharmacy, Second Military Medical University, Shanghai, 200433 China

Retraction to: *Cell Death and Disease* (2017) 8:e3037–e3037 10.1038/cddis.2017.394, published online 07 September 2017

The Editors have retracted this article because of concerns regarding the figures. Specifically, there appears to be overlap between Figure 1C, D, actin controls have been reused in Figures 2C, 4D and 5A, and Fig. 7C appears to be replicated from Fig. 6C of a different article by the same group of authors reporting a different experiment [1]. In addition, there is a concern that the quantifications in this article and [1], e.g. between the histograms in Fig. 1B, C and D in this article and in Fig. 1B, C and D in [1], have very different values for identical experiments, and also have very small standard deviations.

The Editors therefore no longer have confidence in the accuracy of the reported data and the conclusions of the article.

Authors Xiao Chen and Jiacan Su do not agree to this retraction. Authors Xin Zhi, Liehu Cao, Weizong Weng, Honggang Hu, Chao Liu, Qingjie Zhao, Qirong Zhou and Jin Cui have not responded to any correspondence from the Editor or Publisher about this retraction. The Publisher has not been able to obtain a current email address for author Panpan Pan.
